# AI as a reflective partner in EFL speaking: a qualitative study informed by cyborg psychology

**DOI:** 10.3389/fpsyg.2026.1786617

**Published:** 2026-03-23

**Authors:** Hesham Aldamen, Mohamad Almashour, Mutasim Al-Deaibes, Rami Alsharefeen

**Affiliations:** 1 Department of English Language and Literature, The University of Jordan, Amman, Jordan; 2 Department of English, American University of Sharjah, Sharjah, United Arab Emirates; 3 Academic Affairs, Rabdan Academy, Abu Dhabi, United Arab Emirates

**Keywords:** AI-mediated speaking, cyborg psychology, EFL learners, emotional regulation, future self-continuity, language learner identity, qualitative research, reflective learning

## Abstract

**Introduction:**

Speaking anxiety and limited opportunities for spontaneous English interaction remain persistent constraints for many EFL learners in Middle Eastern university settings. This interpretive qualitative study examines how a reflection-enhanced AI speaking design shapes learners' affect, motivation, and future self-concepts.

**Methods:**

Twenty-five Jordanian undergraduates completed a 12-week module in which they practiced weekly speaking with ChatGPT in voice mode (official interface; model label recorded at the time of use). A fixed system prompt configured a supportive, non-judgmental speaking partner, introduced the weekly topic, and triggered two brief in-task reflective questions. Data comprised weekly reflective journals, semi-structured interviews, and saved interaction transcripts.

**Results:**

Using reflexive thematic analysis, we identified three patterned orientations in learners' accounts and interaction traces: initial curiosity with cautious engagement, increasing emotional regulation and psychological safety during speaking, and strengthened future-oriented identity work linking present effort to imagined competent English-speaking selves.

**Discussion:**

Interpreted through cyborg psychology and complementary motivational frameworks, these findings suggest that embedding reflection into human-AI dialogue can support willingness to communicate and identity-related development alongside speaking practice.

## 1 Introduction

Developing oral communication skills remains one of the most persistent challenges for English as a Foreign Language learners in the Middle East, where exposure to English beyond the classroom is limited and instructional practices often emphasize accuracy over meaningful communication. Jordanian university students, in particular, frequently report low speaking confidence, heightened anxiety, and reluctance to engage in spontaneous oral activity (Al-Saidat et al., 2023). These difficulties are consistent with broader findings showing that foreign language anxiety negatively affects learners' willingness to communicate and their speaking performance (Horwitz et al., 1986; Teimouri et al., 2019). Consequently, speaking practice in many regional contexts is often shaped by a fear of evaluation and performance pressure, which restricts opportunities for extended oral engagement. In Jordanian higher education, oral participation can be shaped by face concerns, exam-oriented classroom routines, and limited access to low-stakes English interaction outside class, which may intensify avoidance and constrain opportunities for confidence building.

Recent developments in artificial intelligence have created new possibilities for supporting EFL speaking practice. Conversational AI tools offer accessible, low-pressure environments that may help learners reduce fear of judgment and speaking anxiety (Ding and Yusof, 2025; Wang et al., 2024; Saptiany et al., 2025). These tools can increase opportunities for practice, provide responsive interaction, and allow learners to engage in speaking activities beyond classroom constraints. However, much of the existing research has focused on linguistic outcomes or task performance, with comparatively limited attention to the psychological and motivational processes that influence learners' engagement, confidence, and long-term development. As a result, AI-mediated speaking is often conceptualized primarily as a practice or feedback mechanism, rather than as a psychologically meaningful learning experience (Koç and Savaş, 2024).

This gap is significant given the established role of identity, emotion, and future-oriented motivation in oral communication development. Research has shown that a vivid Ideal L2 Self is associated with higher enjoyment and lower anxiety among Arab EFL learners (Fathi and Mohammaddokht, 2021). Psychological studies further indicate that stronger future self-continuity is linked to persistence, long-term planning, and reduced avoidance behaviors (Hershfield, 2011; Ersner-Hershfield et al., 2023). When learners perceive a clear connection between their present efforts and their future selves as competent English users, they are more likely to invest effort and remain engaged in speaking activities despite discomfort. These findings highlight the importance of structured opportunities for reflection and identity-oriented engagement, particularly in contexts characterized by high anxiety and limited exposure.

Cyborg Psychology offers a complementary perspective on understanding how AI-mediated interactions may support these processes. Pataranutaporn (2024) and Pataranutaporn et al. (2025) conceptualize human-AI interaction as a dynamic psychological system in which AI technologies can function as reflective partners that amplify metacognition, curiosity, identity exploration, and long-term thinking. From this perspective, AI systems are not only tools for task completion but can also support reflective dialogue that encourages learners to articulate goals, beliefs, emotions, and future aspirations. These principles are especially relevant to EFL speaking contexts, where self-concept and identity play a central role in shaping willingness to communicate (Bibauw et al., 2019).

Despite the growing use of AI-mediated speaking tools, most existing designs provide limited reflective scaffolding or psychologically informed support for identity development. Learners often engage with AI at a surface level that prioritizes task completion rather than deeper emotional or motivational engagement. Empirical studies have reported improvements in willingness to communicate, reduced anxiety, and increased self-perceived competence when AI tools are systematically integrated into instruction (Wang et al., 2024; Zhang et al., 2024; Alnemrat et al., 2025; Aldamen et al., 2025). Other research indicates that conversational AI can increase fluency, reduce apprehension, and extend speaking opportunities (Ding and Yusof, 2025), while experimental studies suggest that judgment-free AI rehearsal can lower speaking anxiety (Ebadi et al., 2025). Meta-analytic evidence further emphasizes the importance of contextual, instructional, and socio-emotional factors in determining the effectiveness of AI-supported learning (Hou and Min, 2025; Wu, 2024). Nevertheless, many AI-mediated speaking designs continue to emphasize technological personalization over learners' psychological needs, including reflective thinking and identity development (Kaur et al., 2023). Cyborg Psychology underscores this limitation by arguing that AI systems should promote self-reflection, identity exploration, and long-term growth, rather than focusing exclusively on performance outcomes (Pataranutaporn, 2024).

In response to these gaps, the present study examines how guided self-reflection integrated into AI-mediated speaking practice shapes learners' motivational, emotional, and identity-related experiences. Although conversational AI tools have shown potential for reducing anxiety and increasing speaking opportunities (Ding and Yusof, 2025; Ebadi et al., 2025; Wang et al., 2024; Zou et al., 2024), they have rarely been designed to prompt sustained reflection on learners' goals, future identities, or long-term development. Drawing on the L2 Motivational Self-System (Dörnyei, 2009), research on future self-continuity (Hershfield, 2011; Ersner-Hershfield et al., 2023), and the Cyborg Psychology framework (Pataranutaporn, 2024; Pataranutaporn et al., 2025), this study explores learners' experiences with reflection-enhanced AI-mediated speaking over time. In doing so, the study foregrounds interactional and pedagogical design features, including standardized prompt architecture, voice-based turn-taking rules, and the placement of reflective prompts within the interaction itself, as central components of AI-mediated speaking practice.

While research on AI-mediated speaking in EFL contexts has expanded rapidly, most studies conceptualize AI as a practice tool or feedback provider and evaluate its effectiveness using outcome-oriented measures such as fluency, accuracy, anxiety reduction, or willingness to communicate. Less attention has been paid to how AI-mediated speaking functions as a psychologically situated experience, particularly in relation to emotional regulation, identity development, and future-oriented motivation (Arefian et al., 2024). At the same time, research on L2 motivation and identity has consistently emphasized the role of reflective engagement, Ideal L2 Selves, and future self-continuity in sustaining speaking development, yet these constructs are rarely integrated into the design of AI-mediated speaking tasks. Consequently, there remains limited empirical understanding of how reflection-enhanced AI interaction shapes learners' accounts of speaking and becoming L2 users. By examining a longitudinal, classroom-integrated AI speaking module with carefully specified interaction rules and reflective scaffolding, the present study contributes design-oriented insight to Computer-Assisted Language Learning (CALL), understood as the field concerned with how digital technologies mediate and shape second and foreign language learning processes. Addressing this gap, the study adopts a longitudinal, interpretive approach to examine AI-mediated speaking as a reflective pedagogical process, with attention to learners' experiences of anxiety, confidence, agency, and future-oriented identity.

The study addresses the following research questions:

1. How do EFL learners experience reflection-enhanced AI-mediated speaking practice using ChatGPT?
2. How do reflective AI prompts influence learners' motivation, confidence, and willingness to communicate?
3. How do learners describe the role of AI as a reflective partner in shaping their Ideal L2 Selves and future-oriented thinking?

By foregrounding learners' accounts, this study aims to inform the design of AI-mediated speaking environments that support reflective engagement, identity development, and sustained motivational investment alongside opportunities for oral practice.

## 2 Literature review

### 2.1 EFL speaking challenges, affective factors, and interactional constraints

Speaking proficiency is widely regarded as one of the most challenging aspects of English as a foreign language learning because it requires the coordination of linguistic processing, real-time communication, and affective regulation. Early research identifies foreign language anxiety as a distinct construct that negatively influences oral performance and classroom participation (Horwitz et al., 1986). Meta-analytic findings further confirm that anxiety is consistently associated with lower second language achievement, including speaking outcomes (Teimouri et al., 2019).

Willingness to communicate is a strong predictor of actual speaking behavior and is shaped by learners' confidence, anxiety levels, and situational conditions (MacIntyre et al., 2011). In many Middle Eastern EFL contexts, instruction remains teacher-centered and highly structured, which limits opportunities for spontaneous oral interaction. Research conducted in Jordan indicates that speaking anxiety significantly reduces willingness to communicate and constrains participation in oral tasks (Al-Saidat et al., 2023).

Broader psychological perspectives further emphasize the role of internal states in shaping speaking readiness. Self-determination theory highlights autonomy, competence, and relatedness as essential foundations for intrinsic motivation (Deci and Ryan, 2000), while social learning theory underscores the importance of supportive models and low-risk interaction partners in developing oral skills (Bandura, 1977). These perspectives help explain why speaking challenges persist in contexts characterized by limited communicative exposure and strong evaluative pressure. Cyborg psychology offers an additional perspective for understanding how AI-mediated interaction may influence speaking readiness. Building on accounts of distributed cognition and the extended mind (Anna and Bruno, 2025; Farina and Lavazza, 2022; Hutchins, 1995; Clark and Chalmers, 1998) and emerging human-AI interaction work on beliefs about AI (Pataranutaporn et al., 2023), Pataranutaporn (2024) argues that human-AI interaction can shape emotional preparedness, confidence, and cognitive framing. When AI systems are designed to scaffold comfort, curiosity, and reflection, they may help address affective and interactional barriers that constrain speaking engagement (Bibauw et al., 2019).

### 2.2 AI-mediated speaking practice and learner outcomes

Artificial intelligence-supported conversational tools have increasingly been adopted as individualized environments for speaking practice. Empirical research indicates that AI-powered dialogue systems can enhance fluency, accuracy, and communicative confidence when integrated into structured instructional contexts (Hou and Min, 2025). Ding and Yusof (2025) reported that conversational AI improved speaking performance and reduced anxiety, particularly among learners with limited opportunities for oral interaction. Similarly, Ebadi et al. (2025) demonstrated that AI-generated speaking tasks supported anxiety regulation and oral production by enabling repeated, low-pressure rehearsal.

Research also suggests that chatbot design features influence affective responses. Wang et al. (2024) found that variations in chatbot tone, social presence, and conversational structure affected willingness to communicate, speaking anxiety, and perceived competence. Meta-analytic evidence further indicates that contextual and socio-emotional factors play a significant role in moderating the effectiveness of AI-supported learning (Wu, 2024). Reviews of adaptive AI systems highlight increased engagement and autonomy through personalized scaffolding (Kaur et al., 2023). Despite these reported benefits, most AI-mediated speaking interventions prioritize linguistic support, corrective feedback, or extended practice time rather than psychological growth, identity development, or reflective engagement (Koç and Savaş, 2024). This limitation aligns with Pataranutaporn (2024), who argues that human AI systems should support metacognition, reflection, and long-term psychological development. Reflective elements remain largely absent from the design of AI-mediated speaking tools, leaving motivational and identity-related processes underexplored.

### 2.3 Motivation, the ideal L2 self, and speaking development

Motivation plays a central role in successful second language speaking development, particularly in contexts with limited opportunities for authentic communication. The L2 Motivational Self-System (Dörnyei, 2009) provides a widely adopted framework for understanding how future-oriented self-images sustain effort and engagement. Meta-analytic evidence confirms that the Ideal L2 Self is a strong predictor of motivation, persistence, and long-term achievement (Teimouri et al., 2019).

In Middle Eastern EFL contexts, the Ideal L2 Self-interacts closely with affective variables. Learners with more vivid Ideal L2 Selves report higher enjoyment and lower anxiety (Fathi and Mohammaddokht, 2021). Speaking behavior is also shaped by willingness to communicate, which integrates confidence, emotional regulation, and situational affordances (MacIntyre et al., 2011). A clearly articulated Ideal L2 Self can sustain willingness to communicate by providing a sense of meaning and purpose for speaking efforts.

However, many learners have limited opportunities for structured identity exploration or future-oriented reflection. Pataranutaporn (2024) suggests that reflective AI systems may activate internal motivation, support identity development, and strengthen future-oriented thinking. Integrating reflective scaffolding into AI-mediated speaking activities, therefore, represents a promising yet underexplored approach to supporting speaking engagement (Zhang et al., 2024).

### 2.4 Future self-continuity and psychological foundations

Future self-continuity refers to the perceived similarity and emotional closeness between an individual's present self and imagined future self. Research indicates that stronger future self-continuity is associated with greater persistence, long-term planning, and self-regulation (Hershfield, 2011). Experimental studies have further shown that individuals with higher future self-continuity engage in more future-oriented decision-making and exhibit reduced avoidance behaviors (Ersner-Hershfield et al., 2023). In this study, future-oriented thinking refers to learners' capacity to imagine, elaborate, and evaluate personally meaningful future speaking scenarios and to link present actions to those imagined futures.

This construct aligns closely with the Ideal L2 Self, as both emphasize the motivational value of imagining a capable future identity. In speaking contexts, linking present efforts to future communicative competence may reduce anxiety and increase willingness to engage. Reflective activities embedded in AI-mediated interaction may help strengthen this connection (Zou et al., 2024).

Cyborg Psychology complements this perspective by proposing that AI systems can function as reflective partners that support future-oriented identity development. Pataranutaporn (2024) argues that AI can influence how learners conceptualize their abilities, goals, and possible selves through guided reflection and dialogic engagement.

### 2.5 Gaps in AI-mediated reflective learning and identity support

Although AI-mediated speaking tools have demonstrated positive effects on fluency and anxiety reduction, their design typically emphasizes surface interaction, corrective feedback, or task rehearsal (Ding and Yusof, 2025; Ebadi et al., 2025; Wang et al., 2024). Few systems intentionally prompt learners to reflect on their goals, emotions, or future identities.

Meta-analytic findings suggest that socio-emotional and contextual factors significantly moderate the effectiveness of AI-supported learning (Wu, 2024; Hou and Min, 2025). Reviews of adaptive systems further indicate that technological personalization often receives greater emphasis than psychological personalization (Kaur et al., 2023). This pattern highlights the need for AI designs that explicitly address motivational, emotional, and identity-based dimensions of learning.

Research on future self-continuity demonstrates that connection with future capable selves enhances persistence and self-regulation (Hershfield, 2011; Ersner-Hershfield et al., 2023). Cyborg Psychology extends this view by suggesting that AI can support identity development and reflective thinking through sustained, supportive interaction (Pataranutaporn, 2024). Nevertheless, reflective and identity-oriented design remains rare in AI-mediated speaking research, representing a significant empirical gap (Arefian et al., 2024).

### 2.6 Theoretical framework

The theoretical framework guiding this study integrates affective, motivational, and identity-based perspectives in second language learning with emerging insights from human AI interaction. Four theoretical strands inform the analysis: foreign language anxiety and willingness to communicate, the L2 Motivational Self-System, future self-continuity, and Cyborg Psychology.

Foreign language anxiety and willingness to communicate provide the affective foundation. Anxiety inhibits speaking performance and classroom participation (Horwitz et al., 1986), while willingness to communicate reflects learners' readiness to speak by integrating confidence, affect, and situational conditions (MacIntyre et al., 2011). Reflection-enhanced AI-mediated speaking may support emotional regulation by offering a nonjudgmental environment for practice (Saptiany et al., 2025).

The L2 Motivational Self-System (Dörnyei, 2009) constitutes the second component. The Ideal L2 Self represents a future-oriented image of oneself as a competent language user, and meta-analytic evidence confirms its strong influence on motivated behavior and persistence (Teimouri et al., 2019). Reflective prompts embedded in AI-mediated speaking tasks may help learners articulate and reinforce these future-oriented self-images.

Future self-continuity offers a third lens, emphasizing emotional connection with one's future self as a driver of persistence and reduced avoidance (Hershfield, 2011; Ersner-Hershfield et al., 2023). Reflection within AI-mediated interaction may strengthen this continuity by linking present speaking efforts to future competence.

Cyborg psychology provides an overarching perspective connecting these theories to human-AI interaction. Pataranutaporn (2024) conceptualizes human-AI interaction as a psychologically coupled system in which AI can function as a reflective partner that amplifies metacognition, curiosity, identity exploration, and long-term thinking. To strengthen the theoretical grounding of this lens, we also situate cyborg psychology within established accounts of distributed cognition and the extended mind, which argue that cognition and agency can be distributed across people, artifacts, and environments (Anna and Bruno, 2025; Farina and Lavazza, 2022; Hutchins, 1995; Clark and Chalmers, 1998). Empirically, Pataranutaporn et al. (2023) show that priming beliefs about AI can shape perceived trustworthiness, empathy, and effectiveness in human-AI interaction, highlighting how psychological framing can matter for learning-oriented engagement. Within EFL contexts, AI can therefore be designed as a reflective partner that supports learners in recognizing emotions, articulating aspirations, and envisioning future English-speaking selves (Arefian et al., 2024).

These perspectives position reflection-enhanced AI-mediated speaking as a psychologically meaningful pedagogical environment rather than solely a linguistic practice tool.

## 3 Methodology

### 3.1 Research design

This study employed an interpretive qualitative design to examine how Jordanian EFL undergraduates experienced reflection-enhanced AI-mediated speaking with ChatGPT. Guided by cyborg psychology and complementary accounts of distributed cognition and the extended mind, the learner and ChatGPT were conceptualized as a coupled human-AI system in which cognitive, emotional, and identity-related processes emerge through interaction rather than operating as isolated individual variables (Anna and Bruno, 2025; Pataranutaporn, 2024; Farina and Lavazza, 2022; Hutchins, 1995; Clark and Chalmers, 1998).

An interpretive qualitative orientation was adopted because the study aimed to understand how learners made sense of speaking with AI, including their interpretations of anxiety, confidence, and imagined future English-speaking selves. The purpose was not to measure speaking performance gains through experimental comparison, but to explore how learners interpreted the meaning of sustained, reflection-enhanced speaking engagement over time within a designed AI-mediated interaction.

Three experiential aims guided the study:

1. To explore how learners described their experiences during AI-mediated speaking sessions.
2. To examine how learners perceived changes in willingness to communicate, confidence, and future-oriented self-concepts across 12 weeks.
3. To understand how learners interpreted the role of AI as a reflective partner in their speaking development.

To address these aims, multiple qualitative data sources were collected, including reflective journals, semi-structured interviews, and AI interaction logs. The design prioritizes experiential depth and participant meaning making rather than outcome measurement, consistent with qualitative research in AI-supported language learning.

### 3.2 Participants

Participants were recruited from an Advanced Speaking course offered to English majors at a public university in Jordan. Criterion-based purposeful sampling was used to recruit learners who met the study's eligibility criteria and who voluntarily consented to participate. From a class of 60 students, 25 learners met the inclusion criteria and provided written consent. The remaining students either did not meet eligibility criteria and/or chose not to participate; we therefore acknowledge the possibility of self-selection bias, with participants potentially more willing to engage in AI-mediated speaking than non-participants.

Given the longitudinal, multi-source design, each participant contributed twelve reflective journals, one interview, and multiple saved interaction transcripts. This produced a large and information-rich corpus, supporting analytic depth and thematic adequacy for reflexive thematic analysis. Rather than treating saturation as a procedural endpoint, we monitored whether additional data continued to extend, nuance, or challenge the developing themes across sources and weeks.

Eligibility required that participants were in their 3rd or 4th year of the English major and had completed the university's required English language coursework sequence. All participants possessed intermediate English proficiency, verified through successful completion of B1-level coursework within the university's English language program. These criteria ensured that all participants had sufficient linguistic capacity to engage meaningfully.

All participants were native speakers of Arabic who used English as their primary academic foreign language. English exposure outside the classroom was typically limited, and many learners reported that speaking in English in evaluative classroom settings could be face-threatening, a contextual feature relevant to interpreting anxiety regulation and willingness to communicate in this setting.

### 3.3 Pedagogical and technological design

The study was implemented as a 12-week instructional module integrating AI-mediated, audio-based speaking practice. Each participant completed one session per week, resulting in twelve sessions per learner. All sessions were conducted in a university computer lab to ensure consistent technical conditions, including device configuration, audio quality, and interaction procedures. Participants used desktop computers with headsets, and ChatGPT (voice mode) served as the spoken interaction partner.

Each session lasted approximately 30 min in total, including about 20 min of spoken interaction as well as time allocated for pauses and reflective moments. This structure provided sufficient opportunity for speaking and reflection without requiring continuous speech, making it appropriate for learners at the B1 proficiency level.

At the beginning of each session, students pasted a researcher-designed system prompt into a new ChatGPT window with memory disabled (see Appendix A for the complete system prompt used in all sessions). The prompt served three purposes: configuring ChatGPT as a supportive and non-judgmental speaking partner, introducing the weekly speaking topic, and embedding reflective scaffolding that prompted reflection at two predetermined moments during the interaction. Reflective prompts were triggered automatically by the system prompt to ensure consistency across participants and sessions. Because the interaction was turn-based, learners indicated hesitation or difficulty continuing by submitting short textual cues (e.g., “I'm not sure what to say next”) and pressing the Send button to mark turn completion, which triggered supportive encouragement from the AI rather than topic expansion or corrective feedback.

Each session followed a three-stage cycle. First, during the speaking task phase, ChatGPT initiated the interaction with an open-ended question related to the weekly topic (see Appendix B for the full list of weekly speaking topics). Learners responded orally, elaborated with examples, and continued speaking through follow-up prompts. Second, during the reflection-enhanced interaction, two brief reflective moments prompted learners to consider their emotional state, confidence, communication strategies, and perceived development as English speakers. Third, following the session, learners completed a written reflective journal of approximately 200 to 300 words focusing on emotions, perceived progress, confidence, and future-oriented self-images.

This design positioned ChatGPT as both a speaking partner and a reflective agent. The use of standardized prompts, a controlled lab setting, and a longitudinal structure supported consistency and comparability across sessions and participants.

#### 3.3.1 AI configuration and interaction protocol

All AI-mediated interactions were conducted using ChatGPT in voice mode through the official interface. At the time of use, the model label displayed in the interface was “GPT-5.” Because model deployments and naming conventions can change over time, we report the interface label, access modality (voice mode), and the full system prompt used to configure interactional behavior (Appendix A) to support transparency and replication. Each session began in a new chat window with memory disabled. A standardized system prompt defined ChatGPT behavior, topic focus, and the timing of embedded reflective questions.

All technical settings, equipment, and interaction procedures remained constant across sessions. At the end of each session, learners saved the full, unedited interaction transcript. The researcher provided technical support only and did not observe or monitor conversational content.

#### 3.3.2 Prompt design and standardization

The system prompt followed a consistent structure across all twelve sessions, with only the weekly topic changing. The prompt included four components: definition of ChatGPT's supportive persona, introduction of the speaking topic, behavioral interaction guidelines, and two embedded reflective prompts. Students pasted the complete prompt at the start of each session without modification, ensuring standardized AI behavior across time and participants.

#### 3.3.3 Session structure and logistics

All sessions were conducted under identical conditions in the computer lab. Each session consisted of approximately 20 min of AI-mediated spoken interaction followed by reflective journaling. Learners submitted reflective journals and saved interaction transcripts through the university learning management system. Consistent procedures for timing, setup, and data collection supported reliability across the 12-week module.

#### 3.3.4 Student instructions and training

Prior to the first data-generating session, learners were provided with written and in-class orientation to the procedure. This orientation covered how to access ChatGPT voice mode, how to start a new chat and paste the standardized system prompt, how to manage turn-taking (e.g., speaking in short stretches and pausing when thinking), and how to save the full transcript at the end of each session. Learners were also advised not to share personally identifiable information in the chat and to treat the interaction as practice rather than assessment.

Learners completed a brief familiarization practice with a non-study topic to become comfortable with the interface, voice-to-text display, and the embedded reflective prompts. During the module, technical support was limited to interface or device issues; the researcher did not coach content, provide linguistic feedback, or observe sessions, to reduce instructional influence on participants' accounts.

#### 3.3.5 Interaction log collection

Although interactions occurred orally, the ChatGPT interface displayed a complete text transcript of each session within the chat window. Students manually saved the unedited transcripts at the end of each session and submitted them through the learning management system. Transcripts were anonymized prior to analysis. The researcher did not access or monitor conversations during sessions, minimizing observer influence.

#### 3.3.6 Ethical procedures, participant information, and instructional context

All procedures followed the ethical requirements of the university's research ethics committee. Approval was obtained prior to data collection. Students enrolled in the course received written information describing the aims of the study, the nature of the AI-mediated speaking sessions, and the voluntary nature of participation. They were informed that the study examined their experiences of interacting with ChatGPT over 12 weeks and that participation or non-participation would not affect their course grades. No incentives were provided.

Students who provided written consent agreed to allow their reflective journals, interaction transcripts, and interview data to be used for research purposes. They were informed that the researcher would not observe or listen to their AI-mediated conversations during the sessions. They were also assured that all data would be anonymized. Identifying details were removed during transcription, and pseudonyms such as P01 or P02 were used when excerpts appeared in the findings.

### 3.4 Data analysis

Qualitative data were analyzed using reflexive thematic analysis following Braun and Clarke's (2006) six-phase procedure. Analysis was inductive and focused on identifying patterned meanings across learners' accounts of reflection-enhanced AI-mediated speaking.

First, all reflective journals, interview transcripts, and AI interaction logs were read repeatedly to achieve familiarity with the data. Second, initial codes were generated inductively using NVivo software to organize and manage the qualitative data, with attention to participants' descriptions of emotional experiences, confidence, anxiety regulation, engagement with reflective prompts, and perceptions of their future English-speaking selves. Third, codes were examined and clustered to identify candidate themes that captured shared experiential patterns across participants.

For the AI interaction transcripts, analysis focused on interactional traces relevant to the study's psychological constructs. The unit of analysis was an interactional episode (i.e., a sequence of turns surrounding the opening prompt, moments of difficulty, or the two embedded reflective questions). Transcripts were segmented by session and episode, then coded in NVivo for features such as turn expansion (short vs. extended turns), pause/hesitation markers, self-repair and restarting, explicit emotion labeling, persistence after difficulty, and uptake of reflective prompts. These episode-level codes were used to corroborate, qualify, or problematize interpretations emerging from journals and interviews via case-by-theme matrices, supporting triangulation without treating log data as standalone quantitative performance measures.

In the fourth phase, candidate themes were reviewed in relation to the full dataset to ensure internal coherence and clear distinctions between themes. Fifth, themes were refined, defined, and named to represent how learners experienced AI-mediated speaking as a reflective, emotional, and motivational process over time. In the final phase, themes were synthesized and interpreted in relation to the research questions, emphasizing learners' meaning making rather than frequency counts or outcome-based comparison.

### 3.5 Trustworthiness and rigor

Trustworthiness was supported through triangulation of qualitative data sources, including reflective journals, semi-structured interviews, and AI interaction logs. Analytic rigor was further enhanced through iterative theme development, with themes reviewed and refined across multiple analytic cycles to ensure coherence and clear analytic boundaries. Throughout the analysis, reflexive discussion was used to examine assumptions and maintain sensitivity to participants' perspectives.

Positionality and reflexivity were treated as core components of analytic trustworthiness. The primary researcher designed and implemented the instructional module and also conducted the analysis, which created a potential investment in positive outcomes. To manage this, participation was explicitly voluntary and not tied to course grades; sessions were completed without researcher observation; and reflexive memoing was used throughout coding to surface assumptions, track analytic decisions, and actively seek alternative interpretations, including instances of hesitation, struggle, or constrained engagement.

Member checking was not conducted. Given the interpretive commitments of reflexive thematic analysis, we did not treat participants as validators of theme “accuracy”; however, we acknowledge that the absence of member checking limits opportunities to negotiate meaning with participants and may have reduced visibility of dissenting interpretations. This limitation is addressed explicitly in the Limitations section.

Thick description and representative excerpts were employed to ground interpretations in learners' accounts of reflection-enhanced AI-mediated speaking. Rather than aiming for statistical generalization, the study seeks theoretical transferability to comparable EFL contexts in which speaking anxiety, limited communicative exposure, and AI-supported reflective practice are salient.

### 3.6 Ethical considerations

The study received institutional ethics approval prior to data collection and adhered to established ethical standards for educational research. Participation was voluntary, and all participants provided informed consent. Learners were informed that participation or non-participation would not affect course evaluation.

All data were anonymized prior to analysis, and pseudonyms were used in reporting. AI-mediated speaking sessions were conducted without researcher observation to protect privacy. Interaction transcripts, reflective journals, and interview data were stored securely and accessed only by the research team.

## 4 Findings

The analysis identified three superordinate themes that captured how learners made sense of their experiences during the 12-week reflection-enhanced AI-mediated speaking module: Wonder, Wellbeing, and Wisdom. These themes were developed through reflexive thematic analysis of weekly reflective journals, semi-structured interviews, and AI interaction logs. The three data sources played complementary roles in the analysis. Reflective journals provided contemporaneous accounts of learners' emotional reactions, self-perceptions, and evolving confidence. Semi-structured interviews offered retrospective interpretation and sense-making across time. AI interaction logs documented interactional behavior during speaking, including pauses, hesitation, persistence, and responses to reflective prompts. These sources allowed examination of how emotional, motivational, and identity-related processes unfolded during AI-mediated speaking.

Theme naming was guided by analytic fit rather than rhetorical elegance. The labels Wonder, Wellbeing, and Wisdom are used as interpretive shorthand for three recurring orientations in the dataset that emerged through iterative coding and memoing. We considered more descriptive alternatives (e.g., hesitation-to-curiosity, anxiety regulation, future-self articulation); to foreground analytic precision, we therefore retain the W-labels as memorable umbrella terms while adding descriptive qualifiers in the subsection headings and definitions. Wonder foregrounds early curiosity alongside hesitation and uncertainty; Wellbeing captures in-task emotion regulation and psychological safety during speaking; and Wisdom captures learners' future-oriented sense-making, including resilience and imagined L2 selves. The alliterative naming is intended to improve readability and does not imply a predetermined framework or a fixed developmental sequence.

In developing these themes, we actively searched for divergent or disconfirming accounts. Sustained disengagement or persistent negative evaluations of the AI were not prominent in the journals and interviews; instead, negative experiences were typically episodic (e.g., early-session nervousness, moments of struggle, and occasional interactional breakdowns) and were interpreted by participants as part of learning to persist. We therefore treat the predominantly positive tenor of accounts as an empirical feature of this dataset while also acknowledging, in the Limitations section, the possibility of social desirability and self-selection shaping what participants reported.

### 4.1 Wonder (hesitation-to-curiosity shift)

Learners' initial engagement with AI-mediated speaking was marked by a combination of hesitation, uncertainty, and emerging curiosity. In early reflective journals, many participants described feeling self-conscious and unsure how to interact with the AI. P3 wrote, “I felt shy in the beginning because I did not know how the AI would respond or if it would judge my English.” Similarly, P8 noted, “At first I was careful with every sentence because I was not sure if I was doing it right.” These accounts suggest that early hesitation was linked to unfamiliarity with the interactional context rather than perceived linguistic inability.

AI interaction logs from the first sessions supported these journal accounts. Learners frequently produced short turns, extended pauses before responding, and incomplete utterances followed by restarts. In several transcripts, learners answered the opening prompt with a single clause, paused for several seconds, and then either reformulated or added brief elaboration. These interactional features indicate cautious engagement and monitoring of performance during initial encounters with the AI.

Alongside hesitation, curiosity emerged quickly. Reflective journals documented surprise at the AI's voice, pacing, and responsiveness. P12 wrote, “The AI did not make me feel wrong, and this helped me relax.” P5 commented, “I was surprised that it waited for me and did not interrupt.” This sense of being given time and space appeared to reduce initial apprehension.

Changes were also visible in later interaction logs. Learners produced longer speaking turns, fewer prolonged silences, and more spontaneous elaboration. In several transcripts, learners began to extend responses with personal examples or explanations without explicit prompting. Interviews confirmed this shift in orientation. P7 explained, “When it spoke, it felt like someone was really listening, and this made me want to try more.” P10 similarly stated, “After some weeks, I stopped thinking about the AI and focused on my ideas.” The theme of Wonder captures this early phase of cautious engagement that gradually transformed into curiosity and exploratory talk as learners became familiar with the AI-mediated speaking environment.

### 4.2 Wellbeing (anxiety regulation and psychological safety)

As the module progressed, learners increasingly foregrounded emotional experience and regulation in their accounts of AI-mediated speaking. Reflective journals frequently documented reductions in anxiety and growing emotional stability. P9 wrote, “When the AI asked how I was feeling, I realized I was less nervous than I expected.” P16 noted, “I feel calmer now when I speak, even if I make mistakes.” These entries suggest that learners became more aware of their emotional states and more capable of managing discomfort during speaking.

AI interaction logs provided evidence that emotional noticing occurred within the interaction itself. Following reflective prompts that invited learners to comment on feelings or difficulty, learners often paused and explicitly labeled emotional states such as nervousness, confusion, or growing confidence. In many cases, these moments were followed by resumed speaking with longer turns and fewer interruptions. For example, several transcripts show learners acknowledging anxiety and then continuing to elaborate their ideas rather than terminating the interaction.

Interview data further illuminated how learners interpreted these experiences. P15 stated, “At first I was nervous, but now I feel more stable when I speak.” P4 explained, “Because the structure is always the same, I feel safe and I know what to expect.” Learners consistently attributed emotional ease to the predictable interactional design and the absence of evaluation. Importantly, participants emphasized emotional comfort and readiness to speak rather than improvement in linguistic accuracy. P19 remarked, “My grammar is not perfect, but I feel okay to speak.” The theme of Wellbeing captures learners' accounts of emotional regulation, reduced anxiety, and increased readiness to engage during AI-mediated speaking.

### 4.3 Wisdom (future-self articulation and resilient agency)

In later stages of the module, learners increasingly connected their speaking experiences to longer-term goals, future identities, and personal growth. Reflective journals documented responses to prompts inviting learners to imagine their future English-speaking selves. P11 wrote, “When the AI asked about my future self, I saw myself speaking confidently in my job.” P20 reflected, “I started to think that English is part of my future, not just a course.” These reflections indicate that speaking practice became linked to broader academic and professional aspirations.

AI interaction logs showed changes in how learners managed difficulty. Rather than abandoning responses, learners often paused, acknowledged struggle, and continued speaking. In multiple transcripts, learners verbalized uncertainty and then persisted, sometimes with supportive prompts from the AI. These interactional patterns suggest increased tolerance of difficulty and sustained engagement.

Interviews elaborated on these developments. P6 stated, “I began to see myself as someone who can communicate.” P18 noted, “English feels more connected to my future now.” Learners frequently described the AI as a supportive partner in this process. P2 remarked, “We built the conversation together.” P14 explained, “Now when I struggle, I do not panic. I wait, I think, and I try again.” P22 added, “The AI helped me believe I can continue even when it is hard.”

The theme of Wisdom captures learners' emerging sense of agency, resilience, and future-oriented confidence. Rather than framing development in terms of mastery or performance gains, learners emphasized persistence, self-trust, and alignment between present speaking efforts and imagined future identities.

## 5 Discussion

This study examined how Jordanian EFL undergraduates experienced 12 weeks of reflection-enhanced AI-mediated speaking in voice mode. The findings show recurring ways in which learners interpreted their engagement over time, organized analytically as Wonder, Wellbeing, and Wisdom. These themes do not represent stages or outcomes. They reflect orientations through which learners understood emotional safety, communicative confidence, and future-oriented identity during sustained human-AI interaction. Beyond the thematic account, the study contributes a replicable interactional architecture for reflection-enhanced AI speaking that foregrounds psychological safety and future-oriented motivation (Dörnyei, 2009; Hershfield, 2011).

The discussion draws on reflective journals, semi-structured interviews, and AI interaction logs to interpret how learners experienced speaking with an AI interlocutor. Journals captured ongoing noticing of emotion and engagement. Interviews provided retrospective interpretation of change over time. Interaction logs documented how reflection, hesitation, and persistence occurred during speaking.

### 5.1 AI voice interaction and psychological safety

Learners described initial hesitation and curiosity when speaking with the AI, particularly during early sessions. In journals and interviews, participants emphasized the absence of judgment and evaluation. They contrasted the AI with teachers and peers, noting that pauses, reformulation, and silence were tolerated. Interaction logs show extended pauses, brief turns, and restarts without interruption or negative feedback. These features appeared to reduce social threat rather than eliminate challenge. This pattern is consistent with work showing that speaking anxiety is intensified by evaluative pressure in EFL classrooms (Horwitz et al., 1986; Al-Saidat et al., 2023) and that AI speaking partners can reduce perceived social threat by offering private, low-stakes practice opportunities (Ding and Yusof, 2025; Ebadi et al., 2025; Saptiany et al., 2025).

Voice-based interaction played a role in shaping learners' perceptions of safety. Learners reported that the AI's steady pacing and responsiveness created a sense of being attended to. Interaction logs show learners gradually producing longer turns and fewer prolonged silences. These interactional patterns align with learners' descriptions of increased comfort and willingness to continue speaking. The findings suggest that voice interaction can support psychological safety by allowing learners to manage time, hesitation, and reformulation without fear of evaluation. Prior studies similarly suggest that interactional design features (e.g., pacing, responsiveness, and persona consistency) shape learners' comfort and engagement with conversational agents (Wang et al., 2024; Saptiany et al., 2025).

### 5.2 Reflection and emotional regulation during speaking

A central finding concerns how reflection was embedded within the speaking interaction. Reflective prompts invited learners to comment on emotional states, difficulty, and confidence during speaking rather than after completing the task. In interaction logs, these prompts were often followed by pauses, explicit emotional labeling, and resumed speech. Learners frequently acknowledged nervousness and continued speaking with greater continuity. Conceptually, this aligns with views of affect as dynamically co-constructed in interaction and as a driver of persistence, not merely an after-the-fact attitude report (MacIntyre et al., 2011; Fathi and Mohammaddokht, 2021).

Reflective journals documented similar processes over time. Learners described noticing emotional shifts and developing strategies for managing anxiety. Interviews reinforced these observations by showing how learners interpreted reflection as stabilizing and supportive. Participants emphasized emotional regulation and comfort rather than linguistic improvement. These findings indicate that emotional awareness can be scaffolded through interactional design rather than treated as a separate reflective activity. In other words, reflective design may operate as a micro-scaffold for self-regulation during speaking rather than as a separate metacognitive add-on (Arefian et al., 2024; Hou and Min, 2025).

### 5.3 Willingness to communicate and communicative confidence

Learners described increased willingness to communicate during the module. Journal entries show growing comfort and readiness to speak. Interviews indicate that learners felt more prepared to initiate and sustain interaction in classroom and everyday contexts. Interaction logs show longer speaking turns and fewer abandoned responses over time. This echoes research positioning willingness to communicate as a key proximal predictor of speaking behavior and as sensitive to moment-to-moment affect and context (MacIntyre et al., 2011), as well as recent AI-speaking studies reporting reductions in anxiety and increases in perceived communicative readiness (Ding and Yusof, 2025; Ebadi et al., 2025).

Importantly, learners framed these changes in emotional and situational terms. They did not attribute increased participation to improved accuracy or fluency. Instead, they emphasized feeling calmer, more in control, and more willing to attempt expression. This pattern supports views of willingness to communicate as sensitive to affective and contextual conditions rather than solely to linguistic competence. The present findings therefore complement outcome-focused studies by showing the experiential pathways through which learners come to risk speaking, including feeling safe enough to pause, restart, and persist.

### 5.4 Identity development and future self-continuity

In later sessions, learners increasingly connected speaking experiences to longer-term goals and future identities. Reflective journals show learners articulating imagined future speaking scenarios in academic and professional settings. Interviews extend these reflections by situating weekly practice within broader personal trajectories. Learners described changes in how they viewed themselves as English users. These patterns align with the Ideal L2 Self literature, where vivid future self-images support sustained effort (Dörnyei, 2009; Teimouri et al., 2019), and with future self-continuity work linking emotional connection to future selves with persistence (Hershfield, 2011; Ersner-Hershfield et al., 2023). At the same time, several weekly prompts explicitly invited identity and future-self reflection (Appendix B); we therefore interpret reported identity development as co-constructed by the pedagogical design rather than as a purely spontaneous by-product of AI interaction.

Interaction logs provide evidence of persistence and continuity during moments of difficulty. Learners often paused, acknowledged struggle, and continued speaking rather than terminating the interaction. These behaviors align with learners' descriptions of increased confidence and resilience. The findings suggest that AI-mediated speaking can support identity-related sense-making by linking present effort to future-oriented self-concepts. This interpretation highlights a design-based mechanism: reflective prompts may help learners translate difficult moments into future-oriented meaning, supporting persistence and self-trust.

### 5.5 Shared agency and a cyborg psychology interpretation

Learners frequently described the AI as a supportive partner rather than a replacement for their own effort. In journals and interviews, participants referred to building the conversation with the AI and receiving support during moments of difficulty. Interaction logs show conversational flow emerging through turn-by-turn coordination, reflective prompts, and learner persistence. Similar to findings in human-AI interaction research, learners' willingness to treat the AI as a collaborative partner may depend on how the system is framed and how trust and empathy are perceived (Pataranutaporn et al., 2023).

From a cyborg psychology perspective, AI-mediated speaking can be understood as a coupled system in which agency is distributed between learner and technology. Meaning-making, emotional regulation, and conversational continuity emerged through interaction rather than through tool use alone. This interpretation shifts attention from efficiency or automation to relational and experiential dimensions of human-AI communication. Framed this way, the analysis resonates with distributed cognition and extended mind accounts, which emphasize that cognitive and motivational work can be distributed across interactional partners and tools (Anna and Bruno, 2025; Farina and Lavazza, 2022; Hutchins, 1995; Clark and Chalmers, 1998), while also acknowledging that cyborg psychology remains an emerging lens whose core articulation is currently dissertation-based (Pataranutaporn, 2024).

## 6 Pedagogical implications

The findings suggest several implications for the design of AI-mediated speaking activities in EFL contexts. Voice-based AI environments can provide psychologically safe spaces for oral practice, particularly for learners who experience anxiety in evaluative classroom settings. Design features such as tolerance of silence, patient pacing, and non-judgmental responses appear central to sustaining engagement and encouraging extended speaking. For contexts where evaluative pressure and limited out-of-class exposure constrain oral participation, designing low-stakes speaking spaces may be particularly consequential (Horwitz et al., 1986; Al-Saidat et al., 2023).

Embedding brief reflective prompts within speaking interactions can further support emotional awareness and regulation. Integrating reflection into the interaction itself allows learners to notice and manage affective states as they speak, rather than treating reflection as a separate, post-task activity. This suggestion is consistent with calls to move beyond performance-only outcomes and to attend to affective scaffolding in AI-supported language learning (Hou and Min, 2025; Wu, 2024).

Interactional design also plays a critical role. Features such as predictable pacing and one-question-at-a-time prompts help preserve learner talk time and support a sense of conversational control, reducing the likelihood of AI dominance during interaction. In practice, persona consistency and interaction pacing can be treated as design variables that educators can tune to match learner comfort and task demands (Wang et al., 2024; Saptiany et al., 2025).

Reflection that invites learners to consider future selves can strengthen motivation and support identity development. Connecting present speaking experiences to imagined academic or professional futures may help learners sustain engagement over time and view speaking practice as part of a longer-term developmental trajectory. Future-oriented reflection can be operationalized with concrete scenario-building prompts that connect present speaking tasks to learners' Ideal L2 Selves and perceived future self-continuity (Dörnyei, 2009; Teimouri et al., 2019; Hershfield, 2011).

More broadly, educators can cultivate cyborg-like learning experiences by combining conversational AI with other technology-mediated environments such as virtual reality role-plays, simulation-based speaking tasks, or mobile language-learning apps. Across these tools, the key is to treat technology as part of a coupled learning system: provide psychologically safe interactional conditions, embed brief reflective checkpoints, and make identity- and goal-relevant futures explicit through guided prompts. This approach encourages learners to experience practice as meaning-making and self-development rather than as isolated drill.

Finally, AI-mediated speaking activities are most effective when integrated intentionally into instruction rather than used as unguided independent practice. Pedagogical value emerges from alignment between interaction design, reflective scaffolding, and learning goals.

## 7 Limitations and future research

This study was conducted in a single institutional and cultural context (Jordan) within one Advanced Speaking course. Accordingly, claims are offered as analytically transferable rather than statistically generalizable, and findings may not extend to settings with different participation norms, access to English interaction, or technology policies. The design was interpretive and did not include a comparison group; therefore, the study does not support causal claims about effectiveness relative to other speaking interventions.

Data sources also impose constraints. Weekly reflective journals and interviews are susceptible to self-report bias and social desirability, particularly because the primary researcher designed the module and served as the course instructor, which may have shaped disclosure within the instructional context. While participation was voluntary and not tied to grades, the absence of member checking limited opportunities to negotiate interpretations with participants. In addition, the participant group may reflect self-selection: from a cohort of 60, only 25 learners met eligibility criteria and consented, and non-participants may have differed in motivation or anxiety.

Interaction transcripts were self-saved by learners, and we could not independently verify the completeness of every transcript. Because voice interaction was captured via the platform's voice-to-text display, transcription accuracy and potential data loss are also possible. Reproducibility is further constrained by the rapid evolution of commercial AI systems: we report the interface access mode and the model label shown (“GPT-5”) and provide the full system prompt, but the underlying model and its behavior may change over time. Finally, cyborg psychology is an emerging interpretive lens, and its primary articulation remains dissertation-based (Pataranutaporn, 2024); we therefore triangulate this lens with established distributed cognition and extended mind frameworks (Anna and Bruno, 2025; Farina and Lavazza, 2022; Hutchins, 1995; Clark and Chalmers, 1998) and treat theoretical claims as provisional.

Several design features may have shaped the specific pattern of findings. Some weekly topics explicitly invited identity and future-self reflection (Appendix B), which may have scaffolded the identity-development accounts reported under Wisdom. Future research could compare alternative topic sets, include non-reflective AI speaking conditions, and examine how different cultural participation norms and technology ecosystems shape psychological safety, emotion regulation, and future-oriented motivation in AI-mediated speaking.

## 8 Conclusion

This study examined how Jordanian EFL undergraduates experienced 12 weeks of reflection-enhanced, AI-mediated speaking practice in voice mode. Using an interpretive qualitative approach informed by cyborg psychology, the study focused on learners' emotional experiences, communicative confidence, and future-oriented identity within a structured human-AI interaction.

The findings indicate that learners initially approached AI-mediated speaking with hesitation, but that consistent persona design, patient pacing, and predictable reflective prompts contributed to a psychologically safe environment. This environment supported reduced anxiety and greater willingness to engage in sustained oral communication. Embedded reflective moments played a central role in helping learners notice emotional states, regulate discomfort, and articulate strategies for managing hesitation.

Over time, learners described shifts that extended beyond immediate affect. They articulated more vivid future-oriented self-images, connected present speaking efforts to longer-term goals, and increasingly viewed the AI as a collaborative partner rather than a neutral tool. These accounts highlight the potential of reflection-enhanced AI-mediated speaking to support identity-related sense-making alongside communicative practice.

The study suggests that intentionally designed AI-mediated speaking environments can complement classroom instruction by supporting emotional safety, engagement, and future-oriented motivation. By foregrounding learners' interpretations, the study contributes qualitative insight into how AI-supported speaking may function as a psychologically meaningful pedagogical space and offers a foundation for future research on reflective design in AI-mediated language learning.

## Data Availability

The raw data supporting the conclusions of this article will be made available by the authors, without undue reservation.
